# Supplementary material for: Emotion Regulation Is Associated with Anxiety, Depression and Stress in Adults with Cerebral Palsy
Source: J Clin Med. 2023 Mar 28;12(7):2527. doi: 10.3390/jcm12072527 (PMC10094904; doi:10.3390/jcm12072527)
Supplement: Supplementary file 1 [file jcm-12-02527-s001.zip › File S1.pdf]

Data Dictionary Codebook

19-01-2023 3:27pm

#	Variable / Field Name	Field Label<br>*Field Note*	Field Attributes (Field Type, Validation, Choices, Calculations, etc.)
**Instrument: Emotion Regulation (emotion_regulation)** Enabled as survey			
1	[record_id]	Record ID	text
2	[consent]	Section Header:<br>I consent to participate in this study	radio<br>1 Yes<br>0 No
3	[thankyou_1]<br>Show the field ONLY if:<br>[consent] = '0'	Thank you for your time!	descriptive
4	[first_name]<br>Show the field ONLY if:<br>[consent] = '1'	First name:	text, Required, Identifier
5	[last_name]<br>Show the field ONLY if:<br>[consent] = '1'	Last name:	text, Identifier
6	[contact]<br>Show the field ONLY if:<br>[consent] = '1'	Contact details (phone or email):	text, Required, Identifier
7	[second_contact]<br>Show the field ONLY if:<br>[consent] = '1'	Second contact details (phone or email):	text, Identifier
8	[age]<br>Show the field ONLY if:<br>[consent] = '1'	How old are you (years)?	text (number), Required
9	[thankyou_3]<br>Show the field ONLY if:<br>[age] < 18 and [age] > 1	Thank you for your time!	descriptive
10	[gender]<br>Show the field ONLY if:<br>[consent] = '1' and [age] >= 18	What is your sex?	radio, Required<br>1 Male<br>2 Female<br>3 Other
11	[post_code]<br>Show the field ONLY if:<br>[consent] = '1' and [age] >= 18	What is your residential postcode?	text (postalcode_australia)
12	[employment_status]<br>Show the field ONLY if:<br>[consent] = '1' and [age] >= 18	What is your employment status?	dropdown<br>1 I am paid to work full-time (35+ hours a week)<br>2 I am paid to work part-time or casually<br>3 I volunteer in an unpaid role regularly<br>4 I am a student<br>5 I am not employed but would like to be<br>6 I am not employed and I am not seeking work currently<br>7 Retired
13	[living_arrangement]<br>Show the field ONLY if:<br>[consent] = '1' and [age] >= 18	What is your current living arrangement?	dropdown<br>1 I live alone (with/without children)<br>2 I live in a share house<br>3 I live in supported accommodation<br>4 I live with my spouse (with/without children)<br>5 I live with my parents<br>6 I live with a family member<br>7 Other: Please specify

14	[living_arrangement_comment]<br><br>Show the field ONLY if:<br>[living_arrangement] = '7'	Please specify other living arrangements	notes
15	[children]<br><br>Show the field ONLY if:<br>[consent] = '1' and [age] >= 18	Do you have any children?	radio<br>0 No<br>1 Yes
16	[support_received]<br><br>Show the field ONLY if:<br>[consent] = '1' and [age] >= 18	Have you received care and/or support services?	dropdown<br>1 I receive/d routine care/services to assist me with my daily living activities<br>0 I have not receive/d any routine care to assist in my daily living activities
17	[support_person]<br><br>Show the field ONLY if:<br>[consent] = '1' and [age] >= 18	Who is your primary support person?	dropdown<br>1 Partner/spouse<br>2 Parent<br>3 Friend<br>4 Sibling<br>5 Case worker/advocate<br>6 I don't have a primary support person<br>7 Other
18	[gmfcs]<br><br>Show the field ONLY if:<br>[consent] = '1' and [age] >= 18	Mobility (Gross Motor Function Classification Scale)	dropdown, Required<br>1 GMFCS Level I - I can walk without limitations (but I have some difficulty with running and jumping)<br>2 GMFCS Level II - I walk with some limitations<br>3 GMFCS Level III - I walk using a hand-held mobility device<br>4 GMFCS Level IV - I use wheeled mobility. I can support my head and trunk<br>5 GMFCS Level V - I use wheeled mobility. I have difficulties holding my head and trunk upright independently
19	[intellect]<br><br>Show the field ONLY if:<br>[consent] = '1' and [age] >= 18	Do you have an intellectual impairment?	dropdown<br>0 No impairment<br>1 Mild<br>2 Moderate<br>3 Severe<br>4 Unknown
20	[thankyou_2]<br><br>Show the field ONLY if:<br>[intellect] = '2' or [intellect] = '3'	Thank you for your time!	descriptive
21	[psych_disorder]<br><br>Show the field ONLY if:<br>[intellect] = '0' or [intellect] = '1' or [intellect] = '4'	Have you ever been diagnosed with a psychological disorder?<br>*e.g. depression, anxiety*	dropdown<br>0 No<br>1 Yes (please specify)
22	[psych_specify]<br><br>Show the field ONLY if:<br>[psych_disorder] = '1'	If yes, please specify	text
23	[psych_age]<br><br>Show the field ONLY if:<br>[psych_disorder] = '1'	If yes, how old were you when you were diagnosed with a psychological disorder?	text (number)

24	[psych_managed1]<br>Show the field ONLY if:<br>[psych_disorder] = '1'	On a scale of 1-5 how well do you feel your psychological symptoms are managed?	radio<br>1 1 - Very well managed - experiencing no symptoms<br>2 2 - Well managed<br>3 3 - Sometimes well managed<br>4 4 - Not well managed<br>5 5 - Very poorly managed
25	[cp_type]<br>Show the field ONLY if:<br>[intellect] = '0' or [intellect] = '1' or [intellect] = '4'	Primary motor type of cerebral palsy?	dropdown, Required<br>0 Left hemiplegia/monoplegia<br>1 Right hemiplegia/monoplegia<br>2 Diplegia<br>3 Triplegia<br>4 Quadriplegia<br>5 Dyskinesia - mainly athetosis<br>6 Dyskinesia - mainly dystonia<br>7 Ataxia<br>8 Hypotonia<br>9 Mixed type (please specify)<br>10 Unknown
26	[mixed_type_specify]<br>Show the field ONLY if:<br>[cp_type] = '9'	Please specify:	text
27	[cfcs]<br>Show the field ONLY if:<br>[intellect] = '0' or [intellect] = '1' or [intellect] = '4'	Ability to communicate (using any technique e.g. speech, communication aid etc.)	dropdown<br>1 Effective communicator with unfamiliar and familiar people (CFCS Level I)<br>2 Effective but slower communicator with familiar and unfamiliar people (CFCS Level II)<br>3 Effective communicator with familiar people (CFCS Level III)<br>4 Inconsistent communicator with familiar people (CFCS Level IV)<br>5 Seldom effective communicator even with familiar people (CFCS Level V)<br>6 Unknown
28	[speech]<br>Show the field ONLY if:<br>[intellect] = '0' or [intellect] = '1' or [intellect] = '4'	Ability to speak	dropdown<br>1 Speech is not affected by motor disorder<br>2 Speech is imprecise but usually understandable to unfamiliar listeners<br>3 Speech is unclear and not usually understandable to unfamiliar listeners out of context<br>4 No understandable speech not speak
29	[communication_aid]<br>Show the field ONLY if:<br>[intellect] = '0' or [intellect] = '1' or [intellect] = '4'	Do you use an augmentative and alternative communication system?	dropdown<br>0 No<br>1 Yes
30	[tech_type]<br>Show the field ONLY if:<br>[communication_aid] = '1'	Is it:	radio<br>0 Low tech (gestures, sign language, communication board)<br>1 High tech (voice output communication system)
31	[access_aid]<br>Show the field ONLY if:<br>[tech_type] = '1'	How do you access it?	dropdown<br>0 Direct<br>1 Eye-gaze<br>2 Switch scanning<br>3 Other (please specify below)

32	[other_aid_specify]<br><br>Show the field ONLY if:<br>[access_aid] = '3'	Please specify:	text
33	[ders]<br><br>Show the field ONLY if:<br>[intellect] = '0' or [intellect] = '1' or [intellect] = '4'	Please indicate how often the 36 statements apply to you by selecting the appropriate response	descriptive, Required
34	[ders_1]<br><br>Show the field ONLY if:<br>[intellect] = '0' or [intellect] = '1' or [intellect] = '4'	1. I am clear about my feelings	dropdown, Required<br>1 Almost never<br>2 Sometimes<br>3 About half the time<br>4 Most of the time<br>5 Almost always<br><br>Custom alignment: RH
35	[ders_2]<br><br>Show the field ONLY if:<br>[intellect] = '0' or [intellect] = '1' or [intellect] = '4'	2. I pay attention to how I feel	dropdown, Required<br>1 Almost never<br>2 Sometimes<br>3 About half the time<br>4 Most of the time<br>5 Almost always<br><br>Custom alignment: RH
36	[ders_3]<br><br>Show the field ONLY if:<br>[intellect] = '0' or [intellect] = '1' or [intellect] = '4'	3. I experience my emotions as overwhelming and out of control	dropdown, Required<br>1 Almost never<br>2 Sometimes<br>3 About half the time<br>4 Most of the time<br>5 Almost always<br><br>Custom alignment: RH
37	[ders_4]<br><br>Show the field ONLY if:<br>[intellect] = '0' or [intellect] = '1' or [intellect] = '4'	4. I have no idea how I am feeling	dropdown, Required<br>1 Almost never<br>2 Sometimes<br>3 About half the time<br>4 Most of the time<br>5 Almost always<br><br>Custom alignment: RH
38	[ders_5]<br><br>Show the field ONLY if:<br>[intellect] = '0' or [intellect] = '1' or [intellect] = '4'	5. I have difficulty making sense out of my feelings	dropdown, Required<br>1 Almost never<br>2 Sometimes<br>3 About half the time<br>4 Most of the time<br>5 Almost always<br><br>Custom alignment: RH
39	[ders_6]<br><br>Show the field ONLY if:<br>[intellect] = '0' or [intellect] = '1' or [intellect] = '4'	6. I am attentive to my feelings	dropdown, Required<br>1 Almost never<br>2 Sometimes<br>3 About half the time<br>4 Most of the time<br>5 Almost always<br><br>Custom alignment: RH

40	[ders_7]<br>Show the field ONLY if:<br>[intellect] = '0' or [intellect] = '1' or [intellect] = '4'	7. I know exactly how I am feeling	dropdown, Required<br>1 Almost never<br>2 Sometimes<br>3 About half the time<br>4 Most of the time<br>5 Almost always<br>Custom alignment: RH
41	[ders_8]<br>Show the field ONLY if:<br>[intellect] = '0' or [intellect] = '1' or [intellect] = '4'	8. I care about what I am feeling	dropdown, Required<br>1 Almost never<br>2 Sometimes<br>3 About half the time<br>4 Most of the time<br>5 Almost always<br>Custom alignment: RH
42	[ders_9]<br>Show the field ONLY if:<br>[intellect] = '0' or [intellect] = '1' or [intellect] = '4'	9. I am confused about how I feel	dropdown, Required<br>1 Almost never<br>2 Sometimes<br>3 About half the time<br>4 Most of the time<br>5 Almost always<br>Custom alignment: RH
43	[ders_10]<br>Show the field ONLY if:<br>[intellect] = '0' or [intellect] = '1' or [intellect] = '4'	10. When I'm upset, I acknowledge my emotions	dropdown, Required<br>1 Almost never<br>2 Sometimes<br>3 About half the time<br>4 Most of the time<br>5 Almost always<br>Custom alignment: RH
44	[ders_11]<br>Show the field ONLY if:<br>[intellect] = '0' or [intellect] = '1' or [intellect] = '4'	11. When I'm upset, I become angry with myself for feeling that way	dropdown, Required<br>1 Almost never<br>2 Sometimes<br>3 About half the time<br>4 Most of the time<br>5 Almost always<br>Custom alignment: RH
45	[ders_12]<br>Show the field ONLY if:<br>[intellect] = '0' or [intellect] = '1' or [intellect] = '4'	12. When I'm upset, I become embarrassed for feeling that way	dropdown, Required<br>1 Almost never<br>2 Sometimes<br>3 About half the time<br>4 Most of the time<br>5 Almost always<br>Custom alignment: RH
46	[ders_13]<br>Show the field ONLY if:<br>[intellect] = '0' or [intellect] = '1' or [intellect] = '4'	13. When I'm upset, I have difficulty getting work done	dropdown, Required<br>1 Almost never<br>2 Sometimes<br>3 About half the time<br>4 Most of the time<br>5 Almost always<br>Custom alignment: RH

47	[ders_14]<br>Show the field ONLY if:<br>[intellect] = '0' or [intellect] = '1' or [intellect] = '4'	14. When I'm upset, I become out of control	dropdown, Required<br>1 Almost never<br>2 Sometimes<br>3 About half the time<br>4 Most of the time<br>5 Almost always<br>Custom alignment: RH
48	[ders_15]<br>Show the field ONLY if:<br>[intellect] = '0' or [intellect] = '1' or [intellect] = '4'	15. When I'm upset, I believe that I will remain that way for a long time	dropdown, Required<br>1 Almost never<br>2 Sometimes<br>3 About half the time<br>4 Most of the time<br>5 Almost always<br>Custom alignment: RH
49	[ders_16]<br>Show the field ONLY if:<br>[intellect] = '0' or [intellect] = '1' or [intellect] = '4'	16. When I'm upset, I believe that I'll end up feeling very depressed	dropdown, Required<br>1 Almost never<br>2 Sometimes<br>3 About half the time<br>4 Most of the time<br>5 Almost always<br>Custom alignment: RH
50	[ders_17]<br>Show the field ONLY if:<br>[intellect] = '0' or [intellect] = '1' or [intellect] = '4'	17. When I'm upset, I believe that my feelings are valid and important	dropdown, Required<br>1 Almost never<br>2 Sometimes<br>3 About half the time<br>4 Most of the time<br>5 Almost always<br>Custom alignment: RH
51	[ders_18]<br>Show the field ONLY if:<br>[intellect] = '0' or [intellect] = '1' or [intellect] = '4'	18. When I'm upset, I have difficulty focusing on other things	dropdown, Required<br>1 Almost never<br>2 Sometimes<br>3 About half the time<br>4 Most of the time<br>5 Almost always<br>Custom alignment: RH
52	[ders_19]<br>Show the field ONLY if:<br>[intellect] = '0' or [intellect] = '1' or [intellect] = '4'	19. When I'm upset, I feel out of control	dropdown, Required<br>1 Almost never<br>2 Sometimes<br>3 About half the time<br>4 Most of the time<br>5 Almost always<br>Custom alignment: RH
53	[ders_20]<br>Show the field ONLY if:<br>[intellect] = '0' or [intellect] = '1' or [intellect] = '4'	20. When I'm upset, I can still get things done	dropdown, Required<br>1 Almost never<br>2 Sometimes<br>3 About half the time<br>4 Most of the time<br>5 Almost always<br>Custom alignment: RH

54	[ders_21]<br>Show the field ONLY if:<br>[intellect] = '0' or [intellect] = '1' or [intellect] = '4'	21. When I'm upset, I feel ashamed with myself for feeling that way	dropdown, Required<br>1 Almost never<br>2 Sometimes<br>3 About half the time<br>4 Most of the time<br>5 Almost always<br>Custom alignment: RH
55	[ders_22]<br>Show the field ONLY if:<br>[intellect] = '0' or [intellect] = '1' or [intellect] = '4'	22. When I'm upset, I know that I can find a way to eventually feel better	dropdown, Required<br>1 Almost never<br>2 Sometimes<br>3 About half the time<br>4 Most of the time<br>5 Almost always<br>Custom alignment: RH
56	[ders_23]<br>Show the field ONLY if:<br>[intellect] = '0' or [intellect] = '1' or [intellect] = '4'	23. When I'm upset, I feel like I am weak	dropdown, Required<br>1 Almost never<br>2 Sometimes<br>3 About half the time<br>4 Most of the time<br>5 Almost always<br>Custom alignment: RH
57	[ders_24]<br>Show the field ONLY if:<br>[intellect] = '0' or [intellect] = '1' or [intellect] = '4'	24. When I'm upset, I feel like I can remain in control of my behaviours	dropdown, Required<br>1 Almost never<br>2 Sometimes<br>3 About half the time<br>4 Most of the time<br>5 Almost always<br>Custom alignment: RH
58	[ders_25]<br>Show the field ONLY if:<br>[intellect] = '0' or [intellect] = '1' or [intellect] = '4'	25. When I'm upset, I feel guilty for feeling that way	dropdown, Required<br>1 Almost never<br>2 Sometimes<br>3 About half the time<br>4 Most of the time<br>5 Almost always<br>Custom alignment: RH
59	[ders_26]<br>Show the field ONLY if:<br>[intellect] = '0' or [intellect] = '1' or [intellect] = '4'	26. When I'm upset, I have difficulty concentrating	dropdown, Required<br>1 Almost never<br>2 Sometimes<br>3 About half the time<br>4 Most of the time<br>5 Almost always<br>Custom alignment: RH
60	[ders_27]<br>Show the field ONLY if:<br>[intellect] = '0' or [intellect] = '1' or [intellect] = '4'	27. When I'm upset, I have difficulty controlling my behaviours	dropdown, Required<br>1 Almost never<br>2 Sometimes<br>3 About half the time<br>4 Most of the time<br>5 Almost always<br>Custom alignment: RH

61	[ders_28]<br>Show the field ONLY if:<br>[intellect] = '0' or [intellect] = '1' or [intellect] = '4'	28. When I'm upset, I believe there is nothing I can do to make myself feel better	dropdown, Required<br>1 Almost never<br>2 Sometimes<br>3 About half the time<br>4 Most of the time<br>5 Almost always<br>Custom alignment: RH
62	[ders_29]<br>Show the field ONLY if:<br>[intellect] = '0' or [intellect] = '1' or [intellect] = '4'	29. When I'm upset, I become irritated with myself for feeling that way	dropdown, Required<br>1 Almost never<br>2 Sometimes<br>3 About half the time<br>4 Most of the time<br>5 Almost always<br>Custom alignment: RH
63	[ders_30]<br>Show the field ONLY if:<br>[intellect] = '0' or [intellect] = '1' or [intellect] = '4'	30. When I'm upset, I start to feel very bad about myself	dropdown, Required<br>1 Almost never<br>2 Sometimes<br>3 About half the time<br>4 Most of the time<br>5 Almost always<br>Custom alignment: RH
64	[ders_31]<br>Show the field ONLY if:<br>[intellect] = '0' or [intellect] = '1' or [intellect] = '4'	31. When I'm upset, I believe that wallowing in it is all I can do	dropdown, Required<br>1 Almost never<br>2 Sometimes<br>3 About half the time<br>4 Most of the time<br>5 Almost always<br>Custom alignment: RH
65	[ders_32]<br>Show the field ONLY if:<br>[intellect] = '0' or [intellect] = '1' or [intellect] = '4'	32. When I'm upset, I lose control over my behaviours	dropdown, Required<br>1 Almost never<br>2 Sometimes<br>3 About half the time<br>4 Most of the time<br>5 Almost always<br>Custom alignment: RH
66	[ders_33]<br>Show the field ONLY if:<br>[intellect] = '0' or [intellect] = '1' or [intellect] = '4'	33. When I'm upset, I have difficulty thinking about anything else	dropdown, Required<br>1 Almost never<br>2 Sometimes<br>3 About half the time<br>4 Most of the time<br>5 Almost always<br>Custom alignment: RH
67	[ders_34]<br>Show the field ONLY if:<br>[intellect] = '0' or [intellect] = '1' or [intellect] = '4'	34. When I'm upset, I take time to figure out what I'm really feeling	dropdown, Required<br>1 Almost never<br>2 Sometimes<br>3 About half the time<br>4 Most of the time<br>5 Almost always<br>Custom alignment: RH

68	[ders_35]<br><br>Show the field ONLY if:<br>[intellect] = '0' or [intellect] = '1' or [intellect] = '4'	35. When I'm upset, It takes me a long time to feel better	dropdown, Required<br>1 Almost never<br>2 Sometimes<br>3 About half the time<br>4 Most of the time<br>5 Almost always<br><br>Custom alignment: RH
69	[ders_36]<br><br>Show the field ONLY if:<br>[intellect] = '0' or [intellect] = '1' or [intellect] = '4'	36. When I'm upset, my emotions feel overwhelming	dropdown, Required<br>1 Almost never<br>2 Sometimes<br>3 About half the time<br>4 Most of the time<br>5 Almost always<br><br>Custom alignment: RH
70	[dass]<br><br>Show the field ONLY if:<br>[intellect] = '0' or [intellect] = '1' or [intellect] = '4'	Please read each statement and select how much the statement applied to you over the past week	descriptive, Required
71	[dass_1]<br><br>Show the field ONLY if:<br>[intellect] = '0' or [intellect] = '1' or [intellect] = '4'	1. I found it hard to wind down	radio, Required<br>0 NEVER - did not apply to me at all<br>1 SOMETIMES - some of the time<br>2 OFTEN - a good part of the time<br>3 ALMOST ALWAYS - most of the time
72	[dass_2]<br><br>Show the field ONLY if:<br>[intellect] = '0' or [intellect] = '1' or [intellect] = '4'	2. I was aware of dryness of my mouth	radio, Required<br>0 NEVER - did not apply to me at all<br>1 SOMETIMES - some of the time<br>2 OFTEN - a good part of the time<br>3 ALMOST ALWAYS - most of the time
73	[dass_3]<br><br>Show the field ONLY if:<br>[intellect] = '0' or [intellect] = '1' or [intellect] = '4'	3. I couldn't seem to experience any positive feeling at all	radio, Required<br>0 NEVER - did not apply to me at all<br>1 SOMETIMES - some of the time<br>2 OFTEN - a good part of the time<br>3 ALMOST ALWAYS - most of the time
74	[dass_4]<br><br>Show the field ONLY if:<br>[intellect] = '0' or [intellect] = '1' or [intellect] = '4'	4. I experienced breathing difficulty (e.g. excessively rapid breathing, breathlessness in the absence of physical exertion)	radio, Required<br>0 NEVER - did not apply to me at all<br>1 SOMETIMES - some of the time<br>2 OFTEN - a good part of the time<br>3 ALMOST ALWAYS - most of the time
75	[dass_5]<br><br>Show the field ONLY if:<br>[intellect] = '0' or [intellect] = '1' or [intellect] = '4'	5. I found it difficult to work up the initiative to do things	radio, Required<br>0 NEVER - did not apply to me at all<br>1 SOMETIMES - some of the time<br>2 OFTEN - a good part of the time<br>3 ALMOST ALWAYS - most of the time
76	[dass_6]<br><br>Show the field ONLY if:<br>[intellect] = '0' or [intellect] = '1' or [intellect] = '4'	6. I tended to over-react to situations	radio, Required<br>0 NEVER - did not apply to me at all<br>1 SOMETIMES - some of the time<br>2 OFTEN - a good part of the time<br>3 ALMOST ALWAYS - most of the time
77	[dass_7]<br><br>Show the field ONLY if:<br>[intellect] = '0' or [intellect] = '1' or [intellect] = '4'	7. I experienced trembling (e.g. in the hands)	radio, Required<br>0 NEVER - did not apply to me at all<br>1 SOMETIMES - some of the time<br>2 OFTEN - a good part of the time<br>3 ALMOST ALWAYS - most of the time

78	[dass_8]<br><br>Show the field ONLY if:<br>[intellect] = '0' or [intellect] = '1' or [intellect] = '4'	8. I felt like I was using a lot of nervous energy	radio, Required<br>0 NEVER - did not apply to me at all<br>1 SOMETIMES - some of the time<br>2 OFTEN - a good part of the time<br>3 ALMOST ALWAYS - most of the time
79	[dass_9]<br><br>Show the field ONLY if:<br>[intellect] = '0' or [intellect] = '1' or [intellect] = '4'	9. I was worried about situations in which I might panic and make a fool of myself	radio, Required<br>0 NEVER - did not apply to me at all<br>1 SOMETIMES - some of the time<br>2 OFTEN - a good part of the time<br>3 ALMOST ALWAYS - most of the time
80	[dass_10]<br><br>Show the field ONLY if:<br>[intellect] = '0' or [intellect] = '1' or [intellect] = '4'	10. I felt that I had nothing to look forward to	radio, Required<br>0 NEVER - did not apply to me at all<br>1 SOMETIMES - some of the time<br>2 OFTEN - a good part of the time<br>3 ALMOST ALWAYS - most of the time
81	[dass_11]<br><br>Show the field ONLY if:<br>[intellect] = '0' or [intellect] = '1' or [intellect] = '4'	11. I found myself getting agitated	radio, Required<br>0 NEVER - did not apply to me at all<br>1 SOMETIMES - some of the time<br>2 OFTEN - a good part of the time<br>3 ALMOST ALWAYS - most of the time
82	[dass_12]<br><br>Show the field ONLY if:<br>[intellect] = '0' or [intellect] = '1' or [intellect] = '4'	12. I found it difficult to relax	radio, Required<br>0 NEVER - did not apply to me at all<br>1 SOMETIMES - some of the time<br>2 OFTEN - a good part of the time<br>3 ALMOST ALWAYS - most of the time
83	[dass_13]<br><br>Show the field ONLY if:<br>[intellect] = '0' or [intellect] = '1' or [intellect] = '4'	13. I felt down-hearted and blue	radio, Required<br>0 NEVER - did not apply to me at all<br>1 SOMETIMES - some of the time<br>2 OFTEN - a good part of the time<br>3 ALMOST ALWAYS - most of the time
84	[dass_14]<br><br>Show the field ONLY if:<br>[intellect] = '0' or [intellect] = '1' or [intellect] = '4'	14. I was intolerant of anything that kept me from getting on with what I was doing	radio, Required<br>0 NEVER - did not apply to me at all<br>1 SOMETIMES - some of the time<br>2 OFTEN - a good part of the time<br>3 ALMOST ALWAYS - most of the time
85	[dass_15]<br><br>Show the field ONLY if:<br>[intellect] = '0' or [intellect] = '1' or [intellect] = '4'	15. I felt I was close to panic	radio, Required<br>0 NEVER - did not apply to me at all<br>1 SOMETIMES - some of the time<br>2 OFTEN - a good part of the time<br>3 ALMOST ALWAYS - most of the time
86	[dass_16]<br><br>Show the field ONLY if:<br>[intellect] = '0' or [intellect] = '1' or [intellect] = '4'	16. I was unable to become enthusiastic about anything	radio, Required<br>0 NEVER - did not apply to me at all<br>1 SOMETIMES - some of the time<br>2 OFTEN - a good part of the time<br>3 ALMOST ALWAYS - most of the time
87	[dass_17]<br><br>Show the field ONLY if:<br>[intellect] = '0' or [intellect] = '1' or [intellect] = '4'	17. I felt I wasn't worth much as a person	radio, Required<br>0 NEVER - did not apply to me at all<br>1 SOMETIMES - some of the time<br>2 OFTEN - a good part of the time<br>3 ALMOST ALWAYS - most of the time

88	[dass_18]<br>Show the field ONLY if:<br>[intellect] = '0' or [intellect] = '1' or [intellect] = '4'	18. I felt that I was rather touchy	radio, Required<br>0 NEVER - did not apply to me at all<br>1 SOMETIMES - some of the time<br>2 OFTEN - a good part of the time<br>3 ALMOST ALWAYS - most of the time
89	[dass_19]<br>Show the field ONLY if:<br>[intellect] = '0' or [intellect] = '1' or [intellect] = '4'	19. I was aware of the action of my heart in the absence of physical exertion (e.g. sense of heart rate increase, heart missing a beat)	radio, Required<br>0 NEVER - did not apply to me at all<br>1 SOMETIMES - some of the time<br>2 OFTEN - a good part of the time<br>3 ALMOST ALWAYS - most of the time
90	[dass_20]<br>Show the field ONLY if:<br>[intellect] = '0' or [intellect] = '1' or [intellect] = '4'	20. I felt scared without any good reason	radio, Required<br>0 NEVER - did not apply to me at all<br>1 SOMETIMES - some of the time<br>2 OFTEN - a good part of the time<br>3 ALMOST ALWAYS - most of the time
91	[dass_21]<br>Show the field ONLY if:<br>[intellect] = '0' or [intellect] = '1' or [intellect] = '4'	21. I felt that life was meaningless	radio, Required<br>0 NEVER - did not apply to me at all<br>1 SOMETIMES - some of the time<br>2 OFTEN - a good part of the time<br>3 ALMOST ALWAYS - most of the time
92	[support_services]<br>Show the field ONLY if:<br>[consent] = '1'		descriptive
93	[emotion_regulation_complete]	Section Header: *Form Status*<br>Complete?	dropdown<br>0 Incomplete<br>1 Unverified<br>2 Complete
**Instrument: Comments from researchers (comments_from_researchers)**			
94	[research_comments]	Add any important details relating to this study	notes
95	[comments_from_researchers_complete]	Section Header: *Form Status*<br>Complete?	dropdown<br>0 Incomplete<br>1 Unverified<br>2 Complete
